# Predictive and prognostic values of preoperative platelet parameters in patients with gynecological tumors

**DOI:** 10.1002/jcla.23295

**Published:** 2020-03-13

**Authors:** Wei Yang, Ying‐ying Chen, Chen Bi, Kuang‐yi Shu, Man‐li Ye, Fan‐fan Li, Jie Chen, Xiao‐ou Wang, Xiao‐jian Chen, Ming‐hua Jiang

**Affiliations:** ^1^ The Center of Laboratory Medicine The Second Affiliated Hospital and Yuying Children's Hospital of Wenzhou Medical University Wenzhou China

**Keywords:** diagnosis, gynecological tumors, platelet parameters, prognosis

## Abstract

**Background:**

Platelets play a role in tumor cell growth, metastasis, and angiogenesis, and the present study aimed to evaluate diagnostic and prognostic values of platelet parameters in patients with gynecological tumors.

**Methods:**

A total of 1062 women were included. Differences of platelet parameters (platelet count [PLT], plateletcrit [PCT], mean platelet volume [MPV], platelet‐large cell rate [P‐LCR], and platelet distribution width [PDW]) between different categories were analyzed by nonparametric test. The optimal cutoff value was calculated with receiver operating characteristic analysis. Overall survivals were analyzed with Kaplan‐Meier method and log‐rank tests for univariate analysis.

**Results:**

Platelet count and PCT were significantly increased, and MPV and P‐LCR were significantly reduced in malign and benign gynecological tumor groups compared with the controls (*P* < .001); PDW had no significant differences. There were no significant differences in PLT, PCT, MPV, P‐LCR, and PDW between different tumor locations and pathologic types. The optimal cutoff values of PLT, PCT, MPV and P‐LCR were 274, 0.26, 10.08, and 24.8 (AUC: 0.661, 0.643, 0.593, 0.562), and PCT had preferable sensibility and specificity (50.84% and 70.42%) in predicting the presence of gynecological tumors. According to survival analysis, increased PLT (≥274 × 10^9^/L) and PCT (≥0.26), and induced MPV (<10.08 fL) and P‐LCR (<24.8%) were associated with shorter overall survival.

**Conclusions:**

Platelet count, PCT, MPV, and P‐LCR can be used as preferable auxiliary parameters for predicting the presence of gynecological tumors. Increased PLT and PCT, or decreased MPV and P‐LCR indicated a heavier tumor burden and shorter overall survival.

## 1 INTRODUCTION

With rapid population growth and aging worldwide, cancer incidence and mortality are rapidly growing, thus making cancer as the leading cause of death worldwide.^1^ According to the data released by the global cancer database (GLOBOCAN) in 2018, there were more than 1 309 165 new gynecologic cancer patients and 609 377 women died from gynecological cancer, including cervical cancer, uterine cancer, ovarian cancer, vaginal cancer and vulvar cancer, and cervical cancer ranked fourth for both incidence and mortality in female cancer.^2^ Data also showed that almost 90% of cervical cancer occurred in developing country.^3^ So, easily accessible and inexpensive indicators need to be considered for early diagnosis or prognosis of gynecologic tumors.

Platelets are small (2‐4 um), hematopoietic, and anucleate cells released by bone marrow megakaryocytes in the bloodstream,^4^ and were described as the major effectors of several physiological and pathophysiological processes, such as hemostasis, thrombosis, immunological defense mechanisms, and the development of inflammation.^5^ In addition, a growing body of evidence has found that platelets also play a role in tumor cell growth, metastasis, and angiogenesis.^6^, ^7^ Decades of studies indicated that platelet indicators are important prognostic factors in patients with different types of cancer, such as gynecologic cancers,^8^, ^9^, ^10^, ^11^, ^12^, ^13^ lung cancer,^14^, ^15^ gastric cancer,^16^ colorectal cancer,^5^ pancreatic cancer,^17^, ^18^ laryngeal cancer,^19^ and rectal cancer.^20^ Thrombocytosis may precede the diagnosis of malignancy.^21^ Some studies^6^, ^22^ have tried to explore the complex relationship between platelets and tumors, in order to provide a new basis and way for anti‐tumor therapy. However, few studies have explored the relationship between platelet parameters other than blood platelet count (PLT) and gynecologic cancer, or investigate the differences of platelet parameters between malignant and benign tumors. Herein, we intended to assess the distribution of platelet parameters in patients with malignant and benign gynecological tumors, which were compared to healthy controls, in order to provide some basis for diagnosis and prognosis for gynecological tumors.

## 2 MATERIALS AND METHODS

### 2.1 Patients

Anamnesis, demographic, preoperative laboratory, and histopathological data of women who underwent gynecological tumor surgery for the first time in the Second Affiliated Hospital of Wenzhou Medical University between January 2014 and February 2016 from Hospital Information System were collected retrospectively. Patients were excluded if they had concurrent conditions associated with thrombocytosis (such as chronic obstructive pulmonary disease and collagen vascular disease) or other malignancy, or had received neoadjuvant therapy, or had been treated for recurrent disease.

Platelet parameters were recorded on the basis of the first complete blood count (CBC) within 2 weeks before surgery. The patients we included had been confirmed by pathology results. In addition to the location of the tumor, International Federation of Obstetrics and Gynecology (FIGO, 2009) stage and pathologic type of the tumor were recorded. Besides, CBC of the women who did not show anamnesis of tumor, had not found tumor in the imaging examination, and had normal functions of liver and kidney was obtained from the medical examination center of the Second Affiliated Hospital of Wenzhou Medical University between January 2014 and February 2016.

### 2.2 Data analysis and definitions

The level of PLT in adults was normally ranging from 100 × 10^9^/L to 300 × 10^9^/L.^11^ Thrombocytosis was defined as PLT greater than 400 × 10^9^/L.^22^ The range of plateletcrit (PCT) was normally between 0.11 and 0.28, and PCT more than 0.28 was defined as high PCT. mean platelet volume (MPV) normally ranged from 6.5 to 11.0 fL. Platelet distribution width (PDW) ranged normally between 9% and 17%, and PDW more than 17% was defined as high PDW. For FIGO stages, I and II were defined as early stage, while III and IV were defined as advanced stage.^10^, ^12^

### 2.3 Laboratory assay and pathological examination

Patients’ platelet parameters were obtained from the data of routine preoperative examination after admission. Patients were drawn venous blood after admission which would transport to the laboratory within 1 hour and measured by Sysmex XE5000 hematology analyzer (Sysmex). All microscopic slides and immunohistochemistry of tissue excised intraoperatively were reviewed by two pathologists. Computed tomography (CT) scan and lymph node metastasis sites were used to detect metastatic status.

### 2.4 Statistical analysis

The statistical analysis was performed with SPSS statistical software (version 23.0; SPSS, IBM, Inc.). The median and the interquartile ranges (IQRs) were used for descriptive statistics of indexes in non‐normal distribution. The variables that did not show a normal distribution were compared using the Kruskal‐Wallis H test or the Mann‐Whitney *U* test. The optimal cutoff values of PLT, PCT, MPV, and P‐LCR were analyzed by the ROC curves. Overall survivals (OS) were analyzed with the Kaplan‐Meier method and log‐rank tests for univariate analysis. ROC curves and survival analysis were constructed by GraphPad Prism 7 (GraphPad Software, Inc.). All reported *P* values were two‐sided, and a *P‐*value < .05 was considered as statistical significance.

## 3 RESULTS

### 3.1 Baseline characteristics

We analyzed 1062 women finally, including 214 patients with gynecological cancer, 390 patients with benign gynecological tumor, and 458 normal women. The median age of the patients with gynecological cancer was 52.0 (14.3) years (range: 19‐85), and about 86% of the patients were less than 60 years. The main malignant locations were cervix uteri and corpus uteri. On the basis of staging, 13.1% were diagnosed at advanced stage and 86.9% were at early stage. The median age of the benign group was 44.0 (11.0) years (range: 13‐76), and the main benign locations were corpus uteri and ovary. The median age of normal group was 41.0 (20.0) years (range: 13‐87) (Table 1).

**Table 1:** Clinical characteristics of the patients in the study

	Malign group	Benign group	Control group
	Number (%)	Number (%)	Number (%)
No. of patients	214 (100.0)	390 (100.0)	458 (100.0)
Age (years)			
<60	184 (86.0)	382 (98.0)	379 (82.8)
≥60	30 (14.0)	8 (2.0)	79 (17.2)
Tumor location			
Cervix uteri	109 (50.9)	10 (2.6)	/
Corpus uteri	67 (31.3)	285 (73.1)	/
Ovary	34 (15.9)	87 (22.3)	/
Vagina	1 (0.5)	0 (0.0)	/
Vulva	2 (0.9)	4 (1.0)	/
Oviduct	1 (0.5)	4 (1.0)	/
Pathologic type			
Adenocarcinoma	126 (58.9)	/	/
SCC	80 (37.4)	/	/
Leiomyoma	/	285 (73.1)	/
Teratoma	/	59 (15.1)	/
Adenoma	/	29 (7.4)	/
Others	8 (3.7)	17 (4.4)	/
FIGO stage			
Early (I‐II)	186 (86.9)	/	/
Advanced (III‐IV)	28 (13.1)	/	/
PLT (×10^9^/L)			
<300	149 (69.6)	274 (70.3)	420 (91.7)
≥300	50 (23.4)	96 (24.6)	36 (7.9)
≥400	15 (7.0)	20 (5.1)	2 (0.4)
PCT			
<0.28	123 (57.5)	171 (44.8)	347 (75.9)
≥0.28	91 (42.5)	211 (55.2)	110 (24.1)
MPV (fL)			
<12.00	197 (92.1)	363 (95.0)	419 (91.7)
≥12.00	17 (7.9)	19 (5.0)	38 (8.3)
PDW (%)			
<17	202 (94.4)	372 (97.4)	438 (95.8)
≥17	12 (5.6)	10 (2.6)	19 (4.2)

Abbreviations: “/”, absence; MPV, mean platelet volume; PCT, plateletcrit; PDW, platelet distribution width; PLT, platelet count; SCC, squamous cell carcinoma.

### 3.2 Differences of platelet parameters in groups, tumor locations, pathologic types, or FIGO stages

Platelet count and PCT in malign group and benign group were significantly increased than those in normal group, while MPV and P‐LCR were significantly decreased than those in normal group, but there was no significant difference of PDW among three groups (Table 2). Furthermore, there was no significant difference between disparate tumor locations (cervix uteri, corpus uteri, and ovary), or diverse pathologic types of malignant tumors (adenocarcinoma and squamous cell carcinoma), or diverse pathologic types of benign tumors (leiomyoma, teratoma, and adenoma) (Tables 3, 4, 5, 6). The results showed that the PLT in the malign group and benign group was increased and the volume was smaller in comparison with the normal group, and PLT, PCT, MPV, P‐LCR, and PDW were not associated with tumor locations or pathologic types.

**Table 2:** The association between patients and normal women

	Malign group	Benign group	Normal group	*P‐*value
PLT	261.5 (219.0‐314.3)	261.0 (224.5‐309.5)	229.0 (198.0‐268.0)	<.001
PCT	0.26 (0.23‐0.31)	0.27 (0.24‐0.32)	0.24 (0.22‐0.27)	<.001
MPV	10.1 (9.4‐10.8)	10.3 (9.7‐11.0)	10.5 (9.9‐11.2)	<.001
P‐LCR	26.8 (20.8‐32.2)	27.30 (22.7‐32.9)	28.80 (23.4‐34.9)	<.001
PDW	12.3 (10.7‐14.5)	12.0 (10.8‐13.5)	12.3 (11.1‐13.7)	.845

Abbreviations: MPV, mean platelet volume; PCT, plateletcrit; PDW, platelet distribution width; P‐LCR, platelet‐large cell rate; PLT, platelet count.

**Table 3:** The association between platelet parameters and malign tumor location

	Cervix uteri	Corpus uteri	Ovary	*P‐*value
PLT	254.0 (214.5‐301.0)	268.0 (230.0‐316.0)	277.5 (214.3‐376.8)	.098
PCT	0.26 (0.23‐0.29)	0.26 (0.24‐0.32)	0.27 (0.23‐0.34)	.139
MPV	10.1 (9.5‐10.7)	10.2 (9.3‐10.9)	10.0 (8.9‐11.9)	.963
P‐LCR	26.5 (21.1‐30.4)	27.0 (20.7‐33.4)	29.9 (18.9‐40.4)	.517
PDW	12.1 (10.8‐13.5)	12.1 (10.4‐14.4)	15.5 (10.8‐16.2)	.041

Abbreviations: MPV, mean platelet volume; PCT, plateletcrit; PDW, platelet distribution width; P‐LCR, platelet‐large cell rate; PLT, platelet count.

**Table 4:** The association between platelet parameters and benign tumor location

	Cervix uteri	Corpus uteri	Ovary	*P‐*value
PLT	257.5 (230.8‐303.3)	272.0 (228.0‐311.0)	245.0 (220.0‐287.5)	.158
PCT	0.26 (0.23‐0.29)	0.28 (0.24‐0.32)	0.26 (0.23‐0.29)	.113
MPV	10.1 (9.8‐10.4)	10.3 (9.7‐11.0)	10.4 (9.9‐11.1)	.911
P‐LCR	26.3 (22.7‐27.4)	27.1 (22.7‐33.2)	28.6 (22.7‐33.0)	.653
PDW	11.4 (10.8‐12.1)	11.9 (10.8‐13.5)	12.4 (11.1‐13.6)	.148

Abbreviations: MPV, mean platelet volume; PCT, plateletcrit; PDW, platelet distribution width; P‐LCR, platelet‐large cell rate; PLT, platelet count.

**Table 5:** The association between platelet parameters and pathologic type of malignant tumor

	Adenocarcinoma	SCC	*P‐*value
PLT	265.5 (219‐322.5)	252.0 (217‐307.5)	.112
PCT	0.27 (0.24‐0.32)	0.25 (0.22‐0.30)	.051
MPV	10.1 (9.2‐10.9)	10.2 (9.6‐10.6)	.414
P‐LCR	26.8 (20.5‐33.4)	26.6 (21.1‐30.3)	.734
PDW	12.3 (10.7‐15.2)	12.1 (10.6‐13.3)	.589

Abbreviations: MPV, mean platelet volume; PCT, plateletcrit; PDW, platelet distribution width; P‐LCR, platelet‐large cell rate; PLT, platelet count.

**Table 6:** The association between platelet parameters and pathologic type of benign tumor

	Leiomyoma	Teratoma	Adenoma	*P‐*value
PLT	275.0 (228.0‐311.0)	245.0 (219.0‐289.3)	252.0 (219.5‐304.5)	.165
PCT	0.28 (0.24‐0.32)	0.26 (0.23‐0.29)	0.27 (0.23‐0.30)	.143
MPV	10.2 (9.7‐11.0)	10.6 (9.5‐11.2)	10.3 (10.0‐10.8)	.960
P‐LCR	27.1 (22.7‐33.1)	29.2 (21.0‐33.6)	27.8 (23.6‐32.3)	.962
PDW	11.9 (10.8‐13.5)	12.5 (10.6‐13.8)	12.3 (11.5‐13.8)	.187

Abbreviations: MPV, mean platelet volume; PCT, plateletcrit; PDW, platelet distribution width; P‐LCR, platelet‐large cell rate; PLT, platelet count.

Platelet count and PCT in advanced stage group were significantly increased than those in early stage group, while MPV and P‐LCR were notably decreased than those in early stage group. There was no difference in PDW among the groups (Table 7). The results showed that increased PLT and PCT, or decreased MPV and P‐LCR indicated a heavier tumor burden.

**Table 7:** The association between platelet parameters and FIGO stages

	Early stage	Advanced stage	*P‐*value
PLT	253.0 (217.8‐295.0)	342.5 (293.0‐417.00)	<.001
PCT	0.25 (0.23‐0.29)	0.32 (0.27‐0.44)	<.001
MPV	10.2 (9.5‐10.9)	9.4 (8.8‐10.3)	.003
P‐LCR	27.0 (21.1‐32.7)	23.1 (15.9‐28.4)	.008
PDW	12.4 (10.8‐14.4)	11.7 (9.8‐15.7)	.143

Abbreviations: MPV, mean platelet volume; PCT, plateletcrit; PDW, platelet distribution width; P‐LCR, platelet‐large cell rate; PLT, platelet count.

### 3.3 Value of platelet parameters in predicting the existence of malignant tumor

**Figure 1:**
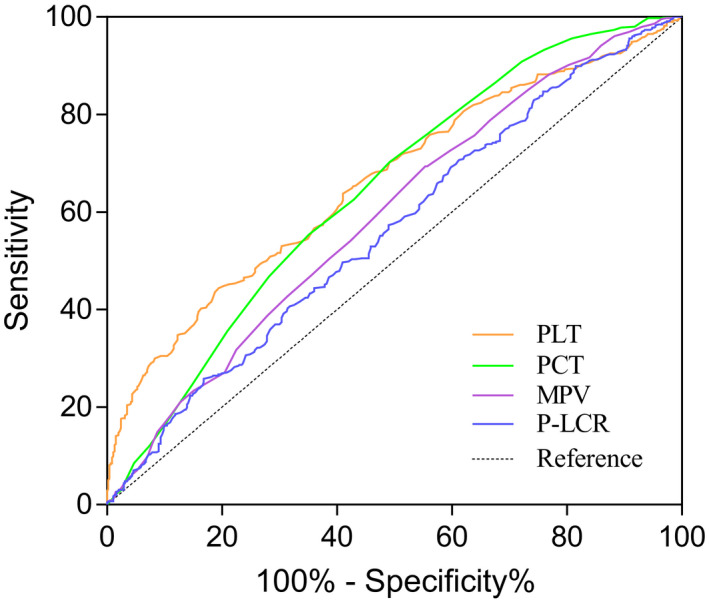
ROC curves for PLT, PCT, MPV, and P‐LCR showing sensitivity and 100%‐specificity% of differential diagnosis of gynecological tumors versus normal group. MPV, mean platelet volume; PCT, plateletcrit; P‐LCR, platelet‐large cell rate; PLT, platelet count; ROC, receiver operating characteristic

On account of similar changes of PLT, PCT, MPV, and P‐LCR in the malign group and benign group, ROC curve analysis was used to verify the predictive ability of PLT, PCT, MPV, and P‐LCR in predicting the presence of gynecological tumors (malignant tumor and benign tumor) (Figure 1). The AUC of PLT was 0.661 (95% CI = 0.632‐0.689, *P* < .001) and the AUC of PCT was 0.643 (95% CI = 0.613‐0.672, *P* < .001) for predicting the presence of gynecological tumors. The AUC of MPV was 0.593 (95% CI = 0.562‐0.622, *P* < .001) and the AUC of P‐LCR was 0.562 (95% CI = 0.531‐0.593, *P* < .001) for predicting the presence of gynecological tumor. The cutoff values of PLT, PCT, MPV, and P‐LCR were 274, 0.26, 10.08, and 24.8, respectively. PLT had the highest specificity with 80.57% (Table 8).

**Table 8:** Area under the curve (AUC), 95% CI, *P‐*value, cutoff value, sensibility, and specificity calculated according to the pathological states in the study group

Variable	AUC	95% CI	*P‐*Value	Cutoff value	Sensibility	Specificity
PLT	0.661	0.632‐0.689	<.001	274	44.37	80.57
PCT	0.643	0.613‐0.672	<.001	0.26	50.84	70.42
MPV	0.593	0.562‐0.622	<.001	10.08	44.63	69.37
P‐LCR	0.562	0.531‐0.593	<.001	24.8	38.76	70.68

Abbreviations: MPV, mean platelet volume; PCT, plateletcrit; P‐LCR, platelet‐large cell rate; PLT, platelet count.

### 3.4 Correlation between platelet parameters and survival of patients with malignancy

**Figure 2:**
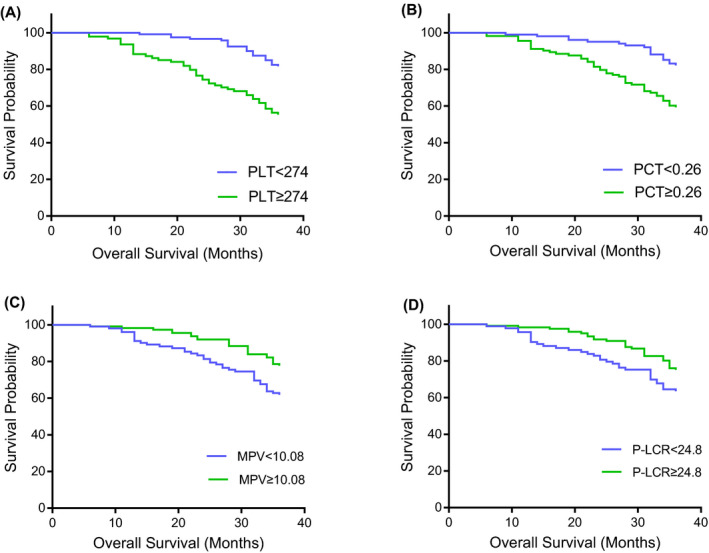
Influence of PLT, PCT, MPV, and P‐LCR on overall survival by Kaplan‐Meier analysis. A, Kaplan‐Meier curves for OS by PLT in gynecological cancer, *P* < .0001. B, Kaplan‐Meier curves for OS by PCT in gynecological cancer, *P = *.001. C, Kaplan‐Meier curves for OS by MPV in gynecological cancer, *P = *.0072. D, Kaplan‐Meier curves for OS by P‐LCR in gynecological cancer, *P = *.0379. MPV, mean platelet volume; OS, overall survivals; PCT, plateletcrit; P‐LCR, platelet‐large cell rate; PLT, platelet count

In survival analysis, the optimal cutoff value of PLT, PCT, MPV, and P‐LCR was determined as 274, 0.26, 10.08, and 24.8, respectively (Figure 1). We found that patients with increased PLT (≥274 × 10^9^/L) and PCT (≥ 0.26) had worse overall survival in comparison with the patients with reduced PLT (<274 × 10^9^/L) and PCT (<0.26), (*P*‐value < 0.0001, hazard ratio [HR]: 3.2, CI: 2.0‐5.4. *P*‐value .0001, HR: 2.6, CI: 1.6‐4.3, respectively) (Figure 2). In addition, patients with reduced MPV (<10.08 fL) and P‐LCR (<24.8%) had worse overall survival in comparison with the patients with increased MPV (≥10.08 fL) and P‐LCR (≥ 24.8%), (*P*‐value = .007, HR: 2.0, CI: 1.2‐3.2. *P*‐value = .038, HR: 1.7, CI: 1.0‐2.8, respectively) (Figure 2).

## 4 DISCUSSION

Since the first study on platelets and tumors a century ago, the relationship between platelets and various malignant tumors or the involvement of platelets in malignant tumor growth and metastasis had emerged one after another.^7^, ^23^, ^24^, ^25^ Nevertheless, a majority of studies were focused on the relationship between the malignancy and the PLT. Other platelet parameters such as PCT, MPV, P‐LCR, and PDW had not been widely studied.

In this study, the median (IQRs) level of PLT of patients with gynecological cancer was 261.5 (219.0‐314.3) × 10^9^/L, and 15% of the patients had thrombocytosis, which was in accordance with previous literature.^8^, ^21^, ^26^ According to the existing studies, the increase in PLT is due to tumor‐secreted cytokines (including interleukin (IL)‐1, IL‐3, IL‐6, and leukemia inhibitory factors) that play a role in stimulating megakaryocyte growth and thrombopoiesis.^21^ But we found that increased PLT occurred not only in gynecological malignant tumors, but also in benign gynecological tumors. It remained unclear whether it was a result of tumor‐secreted cytokines or caused by the bleeding that activated the clotting pathway because of the tumor.

We found that MPV in malign group and benign group (median [IQRs]:10.1 [9.4‐10.8] and 10.3 [9.7‐11.0], respectively) was significantly decreased than those in normal group (median [IQRs]: 10.5 [9.9‐11.2]). The literature on this topic included conflicting results. Temur, et al supported that there was no difference of MPV between patients with endometrial cancer and controls,^27^ while others showed that MPV was higher in patients with endometrial cancer compared with controls.^28^ It was unclear whether the heterogeneous size and structure of the platelet translated into differences in platelet function.^29^ The potential value of MPV in gynecological cancer needs to be further investigated. PCT and P‐LCR were found significantly different between patients and controls. Nevertheless, the association between PCT, P‐LCR, PDW, and gynecological tumor had rarely been investigated in previous research.

A large amount of literature showed that platelets played a certain role in the growth and metastasis of malignant tumors,^6^, ^7^, ^23^, ^25^ accompanied by changes in number and size of platelets.^9^, ^10^, ^28^, ^30^ We found that there were similar changes in the number and size of platelets in patients with gynecological malignant and benign tumors (Table 2), and there was no significant difference between different pathological types and different organs (Table 3). According to the ROC curves (Figure 1), PLT, PCT, MPV, and P‐LCR had preferable specificity in predicting the presence of gynecological tumor. They could be useful parameters for clinical assistant diagnosis. However, the mechanism of changes in the numbers and size of platelet in gynecological tumors is unclear and needs to be further investigated.

It was found that patients with advanced disease had a higher mean preoperative PLT than patients with localized disease, and preoperative thrombocytosis was an independent prognostic indicator for high‐risk patients with stages III to IV endometrial cancer.^31^ Katrin et al supported that thrombocytopenia suggested advanced tumor, higher tumor grade, and higher incidence of serous ovarian cancer.^32^ Our present data (Table 7) agreed with previous studies, and we found that MPV, PCT, and P‐LCR were associated with FIGO stages. High PCT, low MPV, and low P‐LCR indicated worse tumor staging.

The association between the change of platelet parameters and OS of patients with gynecological cancer has been demonstrated in a few studies. Li et al^33^ suggested that thrombocytosis predicted poorer survival in women with advanced stage epithelial ovarian carcinoma. Suttichai et al^9^reported that thrombocytosis is not uncommon in endometrial carcinoma and may reflect adverse prognostic factors. Our data suggested that increased PLT predicted poor prognostic; it was consistent with above research. Moreover, Isa et al^27^ demonstrated that MPV was not found to be associated with prognostic factors or survival. But we found that MPV was lower in patients with gynecological cancer compared to controls, and patients with low MPV (<10.08 fL) showed shorter overall survival. In addition, we found that increased PCT (≥0.26) and reduced P‐LCR (<24.8%) were associated with shorter overall survival, but in the absence of relevant literature, more data are needed to prove it.

The major limitations of this study are as follows: (a) The study was conducted in a single center, (b) the potential for recall error was existed on account of the retrospective study, and (c) information on progression‐free survival was not included in the study because of telephone follow‐up.

In conclusion, the increased PLT and the decreased platelet volume were not only common in gynecological cancer, but also usual in gynecological benign tumors. PLT, PCT, MPV, and P‐LCR were useful auxiliary parameters for predicting the presence of gynecological tumor. In addition, increased PLT and PCT, or decreased MPV and P‐LCR indicated a heavier tumor burden and shorter overall survival.

## ETHICAL APPROVAL

All procedures performed in studies involving human participants were in accordance with the ethical standards of the institutional and/or national research committee and with the 1964 Helsinki Declaration and its later amendments or comparable ethical standards.
